# Comparative genomic analysis of *Babesia duncani* responsible for human babesiosis

**DOI:** 10.1186/s12915-022-01361-9

**Published:** 2022-07-05

**Authors:** Jinming Wang, Kai Chen, Jifei Yang, Shangdi Zhang, Youquan Li, Guangyuan Liu, Jianxun Luo, Hong Yin, Guangying Wang, Guiquan Guan

**Affiliations:** 1 State Key Laboratory of Veterinary Etiological Biology, Key Laboratory of Veterinary Parasitology of Gansu Province, Lanzhou Veterinary Research Institute, Chinese Academy of Agricultural Science, Lanzhou, 730046 Gansu China; 2 Key Laboratory of Aquatic Biodiversity and Conservation, Institute of Hydrobiology, Chinese Academy of Sciences, Wuhan, 430072 China; 3 Department of Clinical Laboratory, The Second Hospital of Lanzhou University, Lanzhou, 730030 China; 4 Jiangsu Co-Innovation Center for the Prevention and Control of Important Animal Infectious Disease and Zoonoses, Yangzhou University, Yangzhou, 225009 China

**Keywords:** De novo assembly, *Babesia duncani*, Babesiosis, Phylogenetic analysis, Adaptive evolution, Invasion

## Abstract

**Background:**

Human babesiosis, caused by parasites of the genus *Babesia*, is an emerging and re-emerging tick-borne disease that is mainly transmitted by tick bites and infected blood transfusion. *Babesia duncani* has caused majority of human babesiosis in Canada; however, limited data are available to correlate its genomic information and biological features.

**Results:**

We generated a *B. duncani* reference genome using Oxford Nanopore Technology (ONT) and Illumina sequencing technology and uncovered its biological features and phylogenetic relationship with other Apicomplexa parasites. Phylogenetic analyses revealed that *B. duncani* form a clade distinct from *B. microti*, *Babesia* spp. infective to bovine and ovine species, and *Theileria* spp. infective to bovines. We identified the largest species-specific gene family that could be applied as diagnostic markers for this pathogen. In addition, two gene families show signals of significant expansion and several genes that present signatures of positive selection in *B. duncani*, suggesting their possible roles in the capability of this parasite to infect humans or tick vectors.

**Conclusions:**

Using ONT sequencing and Illumina sequencing technologies, we provide the first *B. duncani* reference genome and confirm that *B. duncani* forms a phylogenetically distinct clade from other Piroplasm parasites. Comparative genomic analyses show that two gene families are significantly expanded in *B. duncani* and may play important roles in host cell invasion and virulence of *B. duncani*. Our study provides basic information for further exploring *B. duncani* features, such as host-parasite and tick-parasite interactions.

**Supplementary Information:**

The online version contains supplementary material available at 10.1186/s12915-022-01361-9.

## Background

Human babesiosis, caused by the genus *Babesia*, is an emerging and reemerging tick-borne infectious disease that is mainly transmitted by tick bites, blood product transfusion, and congenitally. There is a broad agreement that the main causative agents of human babesiosis are *B. microti*, *B. divergens*, *B. duncani*, *B. crassa*, and *B. motasi* [1–6]. Symptoms of babesiosis include fever, headache, multi-system organ failure, and even death [7, 8]. In recent years, an increasing number of cases of human babesiosis have drawn people’s attention. During the past decades, the majority of knowledge has been obtained about *B. microti*, which is responsible for the majority of *Babesia* species infections in humans throughout the world. Traditionally, other *Babesia* spp. have been neglected because they cause relatively fewer cases in comparison with *B. microti* [9]. Compared with *B. microti*, *B. duncani* is characterized by a rapid increase in parasitemia and severe pathology, with mortality rates of around 95% in infected C3H, A/J, AKR/N, and DBA/1J mice [10, 11]. The recent emergence of significant human babesiosis cases caused by *B. duncani* in the Pacific coast region, as well as the observation of severe and even fatal human cases, has stimulated interest in this enigmatic species, including its biological features, phylogenetic relationships, and adaptive evolution.

As the highest virulent *Babesia* species as-confirmed in animal models (such as in mice and hamsters), *B. duncani* was first reported in a 41-year-old man who contracted human babesiosis in Washington state in 1993 [12]. Since then, additional cases caused by this pathogen were documented in California and Canada [13, 14]. Earlier studies confirmed *B. duncani* in a clade with parasite *B. conradae* isolated from canines in California, based on its phylogenetic analysis targeting the 18S rRNA gene, whereas by targeting the ITS (Internal transcribed spacer) gene, *B. duncani* was placed in a distinct clade from other known *Babesia* spp. [15]. However, those controversial conclusions were recently challenged with the completeness of mitochondrial genome sequencing that placed this parasite in a clade with *T. orientalis* and *T. parva*, infecting buffo and cattle, respectively, but distinct from other *Babesia* spp. and *Plasmodium* spp. [16]. The updated phylogeny-based classification of the Piroplasmida is challenging the previous taxonomic evolutionary analysis. *Babesia* spp. form a polyphyletic group, in terms of their phenotype and life history, which provides valuable information for understanding the taxonomy of the Piroplasmida [17]. The *B. duncani* lineage is classified as *Babesia* sensu lato, suggesting that *B. duncani* belongs to clade III of Piroplasmida [18].

In this context, we use genomic and transcriptomic data derived from *B. duncani* merozoites to assemble a reference genome and to perform a comparative analysis of genomes from apicomplexan parasites. Our analyses provide a better understanding of its evolution and key features correlated with its biology, such as gene family expansion and host cell invasion.

## Results and discussion

### Genome assembly and annotation

**Fig. 1:**
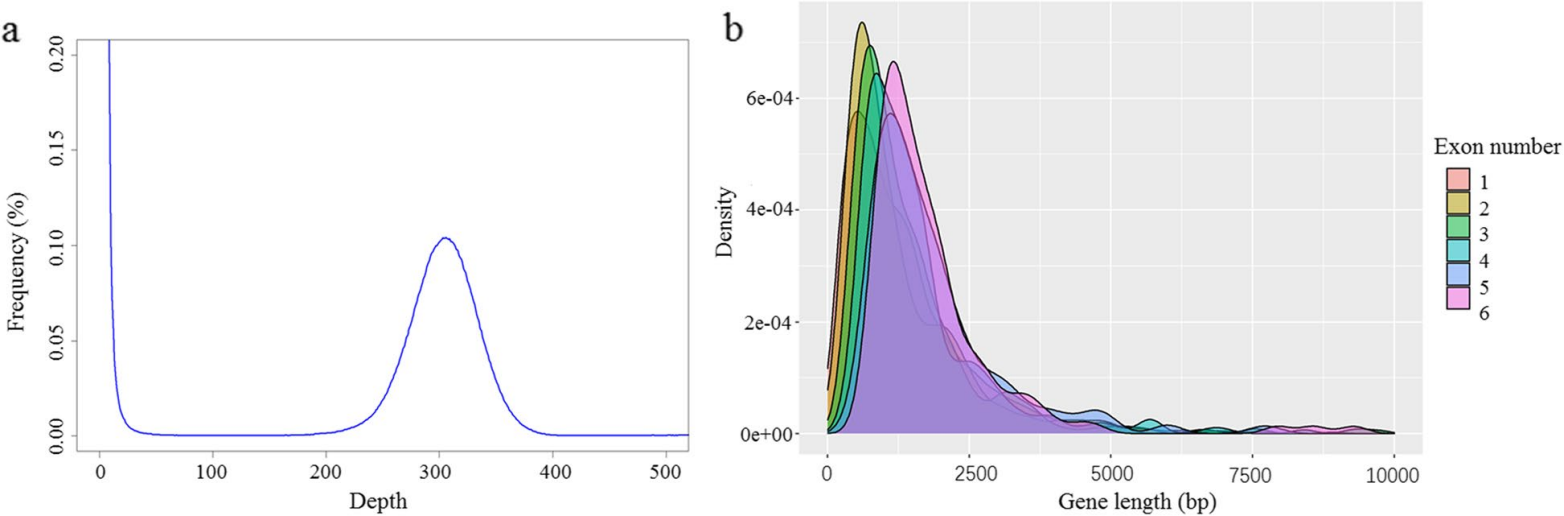
Sequencing depth estimation and density of gene length with variable exon number. **a** Sequencing depth was evaluated using Jellyfish. **b** Gene length increased with exon numbers

**Fig. 2:**
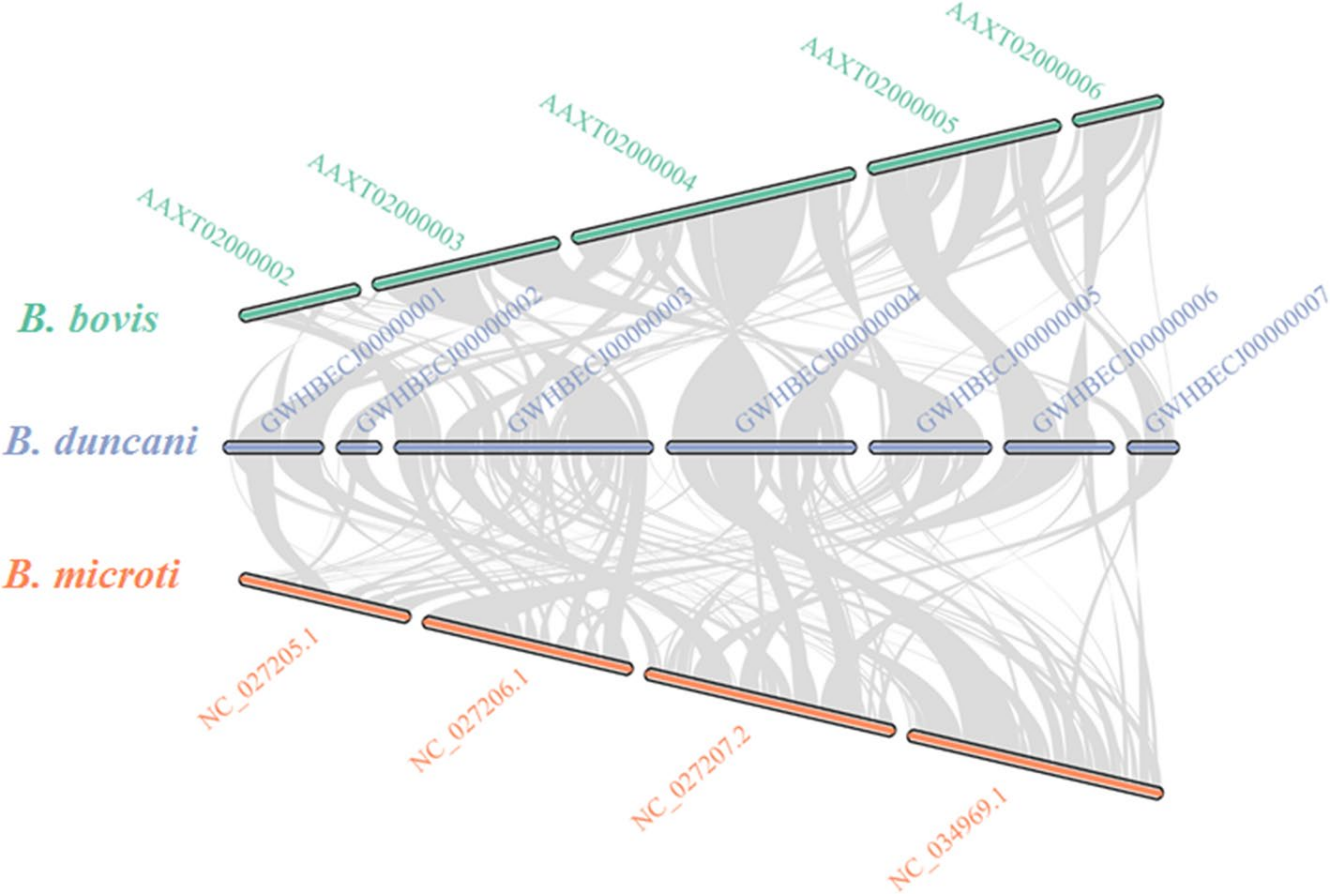
Genome collinearity analysis of *B. duncani* with *B. bovis* and *B. microti*. The collinear gene blocks were determined by MCScanX between genome scaffolds for three *Babesia* spp.

**Fig. 3:**
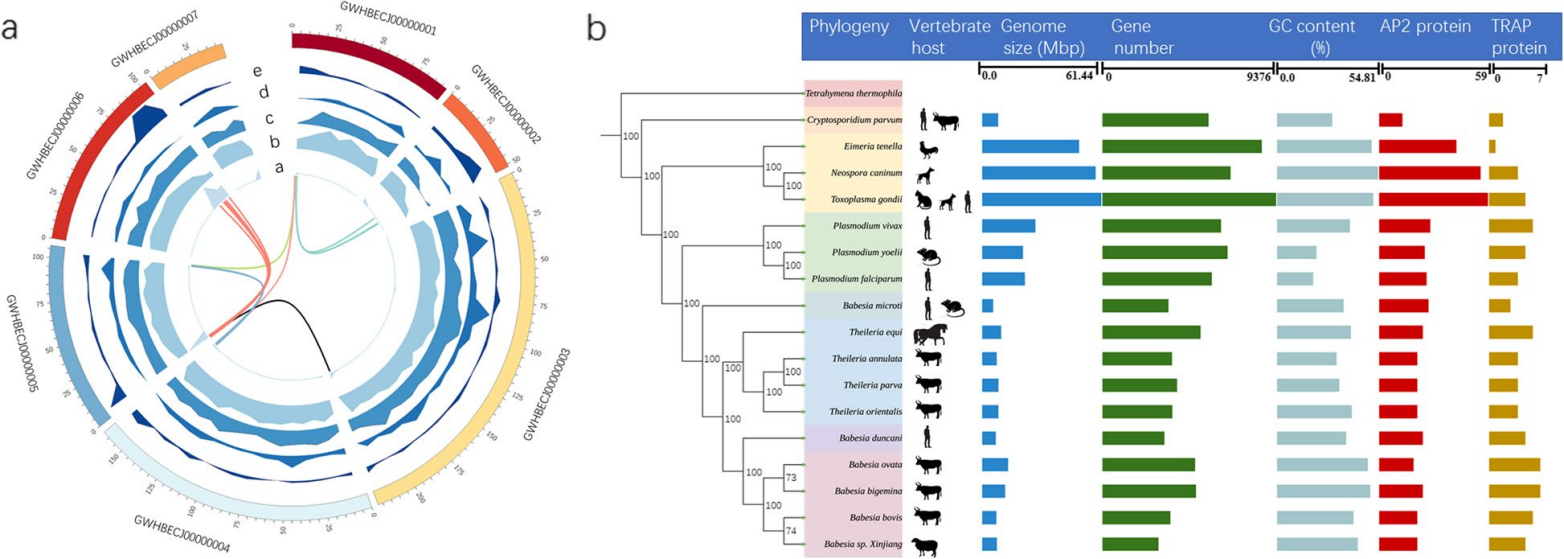
Phylogenetic relationship with other Apicomplexa parasites and critical features of their genomes. **a** Circular plot shows basic features of the *B. duncani* genome assembly. From the outermost to the innermost ring: density of short interspersed elements, levels of gene expression, % of GC content, density of gene model, and gene family of *blf* 1. **b** Maximum likelihood species tree of Apicomplexa parasites was constructed based on a concatenated alignment of 493,035 amino acids from 310 single-copy orthologous genes. The rooting of the tree at *Therahymena thermophila* is based on a previously documented *Babesia* phylogenetic relationship [21]. Bootstrap values were shown on each node

*Babesia duncani* genome was sequenced using ONT and Illumina platforms. Long reads derived from ONT (182,649 reads, median length 25,597 bp, total bases 4.7 Gb) were assembled into the draft assembly, which was further corrected using ONT long reads and Illumina reads (length 150 bp, total paired sequences 9,442,873, total bases 2.2 Gb). The overall coverage of sequencing data was evaluated using Jellyfish v2.3.0 (Fig. 1a) [19]. Eventually, a total length of 7.9 Mb with seven scaffolds was generated, which size is comparable to *B. microti*, *B. bovis*, and *Babesia* sp. Xinjiang, ranging from 6.4 to 8.4 Mb. However, the *B. duncani* genome is the second smallest *Babesia* spp. genome. GC content is similar between *B. microti* and *B. duncani* (Table 1), but lower than other Piroplasm parasites, such as *B. bovis*, *B. bigemina*, and *B. ovata*. *Babesia duncani* genome contains 9.1% repeated sequences, including 8.8% of unclassified repeats and 0.2% simple repeats, and classic transfer RNA genes. The completeness of the genome, evaluated by BUSCO (v5.1.3) using the core apicomplexan dataset (apicomplexa_odb10), was 95.3% [20]. Comparisons of *B. duncani* reference genome with these of *B. bovis* and *B. microti* reveal some common features, including a similar GC content, some degree of collinearity, rearrangements of large fragment, and conservation of gene content (Table 1, Figs. 2, and 3a).

**Table 1:** Genome statistics of *B. duncani* comparison with other reference genomes of *Babesia* spp.

	*B. bigemina*	*B. bovis*	*B. duncani*	*B. microti*	*B. ovata*	*Babesia* sp. Xinjiang
Genome size (Mbp)	13.8	8.2	7.9	6.4	14.5	8.4
N50	2,541,256	1,797,577	1,067,452	1,766,409	2,090,503	533,301
GC (%)	50.63	41.69	37.68	36.17	49.27	43.87
Coding gene numbers	5079	3974	3759	3573	5044	3066
*N*’s per 100 kbp	0	0.01	0.01	1.59	0	591.11
Gene density (gene/Mb)	368	484.6	475.8	558.3	347.9	365
Number of exons per gene	2.6	2.8	2.7	7.7	2.5	3.3
Apicomplast genome						
Genome size (bp)	nd	35,107	34,142	28,657	nd	30,729
GC (%)	nd	22	15.2	14.1	nd	19
No. of genes	nd	58	38	57	nd	57
Mitochondrion genome						
Genome size (bp)	nd	6005	5893	10,547	nd	5767
GC (%)	nd	29.5	31.85	35.2	nd	29.13
No. of genes	nd	8	9	11	nd	9
Completeness evaluation (BUSCO)	96.40%	96.90%	95.30%	94.80%	96.80%	96.20%

*nd* not determined

We predicted 3759 protein-coding genes in the *B. duncani* genome, which is similar in the number of genes to those of other *Babesia* spp., 61.4% of which were proved by RNA-Seq data. Almost half of these genes (1636 genes) were annotated to Gene Ontology (GO) terms (Additional file 1: Table S1). A total of 369 (9.8%) of predicted proteins contained signal peptide sequences. A total of 1625 genes are lacking introns, and the remaining 2134 (56.8%) genes are present with one or several introns. The percentages of genes containing one or even more introns are almost similar in lineage-specific gene families (52/89, 58.4%) and expanded gene families (12/21, 57.1%), whereas a significantly high percentage of multiple introns genes is observed in conserved housekeeping genes (1279/1900, 67%) (*p* < 0.01). Gene length is a positive correlation with the number of exons (Fig. 1b).

### Phylogenetic relationship with other Apicomplexa parasites

There is an agreement that mitochondrial protein-coding sequences are commonly used to investigate the evolutionary and phylogenetic relationships of apicomplexan parasites. Recently, analysis of cytochrome c oxidase subunit I (*CoxI*), cytochrome b (*Cob*) protein sequences, and 18S rRNA revealed that *B. duncani* was defined in a clade with *Theileria* spp. (including *T. orientalis* and *T. parva*), whereas it has a relatively remote phylogenetic relationship with *Babesia* spp .[16, 17]. In addition, to provide a reliable evolutionary position of *B. duncani* and fully analyze the relationship between this parasite with Apicomplexa parasites, 310 single-copy orthologous nuclear genes from 18 species across Apicomplexa were used to reconstruct maximum likelihood phylogenetic trees. Our result is consistent with the previous results, based on the analysis of *CoxI* and *Cob* sequences, that *B. duncani* is ascribed to a new lineage distinct from *B. microti*, *B. bovis*, *Theileria* spp., and *Plasmodium* spp. [16]. It is noted that when *B. microti* is included, *Babesia* spp. are paraphyletic, with sister-group relationships of *B. bigemina* and *B. ovata* with *Babesia* sp. Xinjiang and *B. bovis*. In contrast to the previous results that ascribed a close relationship between *B. duncani* and two *Theileria* spp. (*T. orientalis* and *T. parva*), this parasite falls in the same group as four other *Babesia* spp. (*B. bigemina*, *B. ovata*, *Babesia* sp. Xinjiang, and *B. bovis*), but itself forms a separate clade [16]. Obviously, each species responsible for human babesiosis forms a monophyletic clade. One reason for our results differing from previous reports is limited phylogenetic information on the single or a few genes used to analyze this relationship. Our results provide robust evidence to resolve the position for this human pathogen (Fig. 3b).

**Fig. 4:**
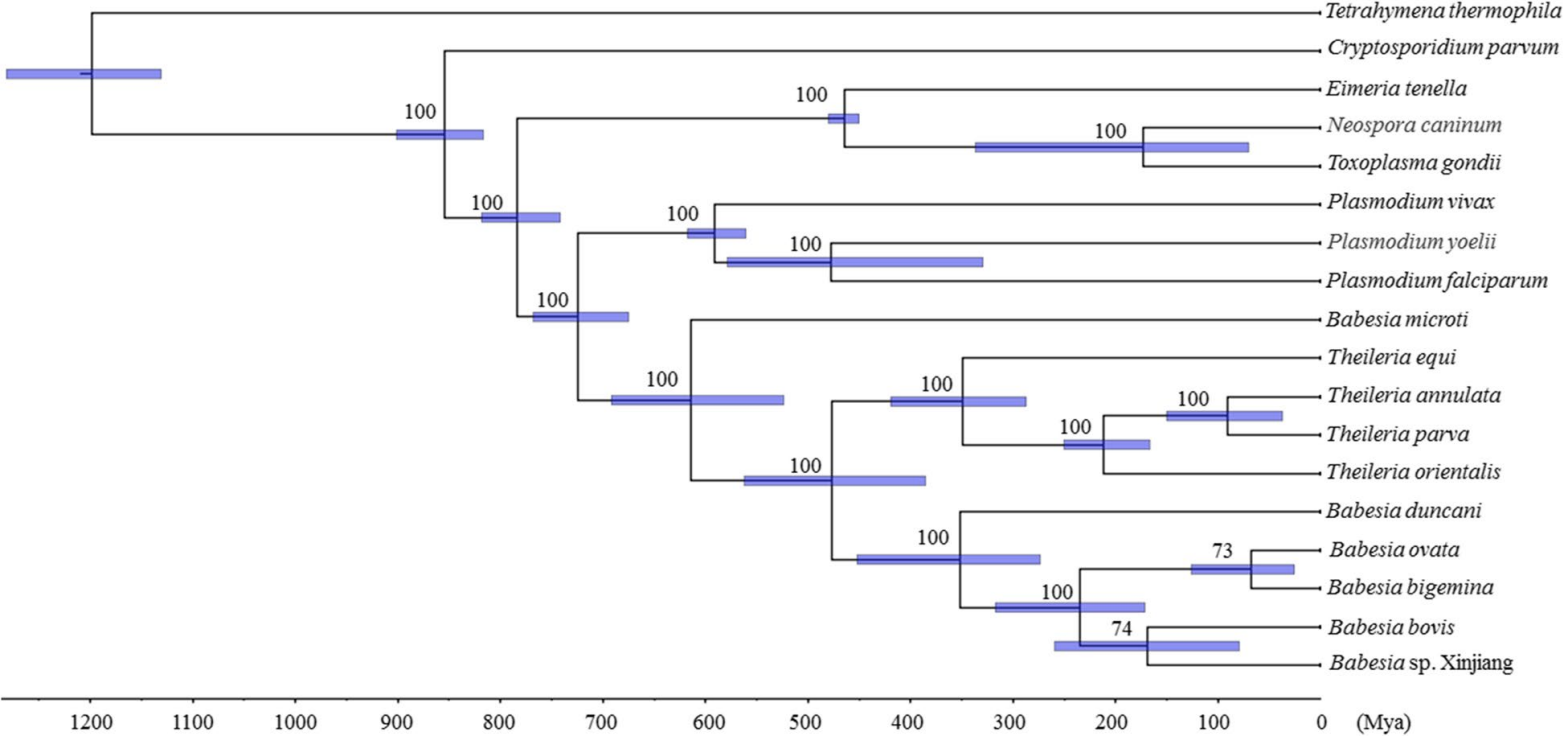
Estimated divergence time across Apicomplexa parasites. Species dates were determined using 310 orthologous groups across 18 Apicomplexa parasites and an outgroup species reference genomes. Three fossil times were included to calibrate the split of these species. Ninety-five percent confidence intervals for each node are shown by heat maps

Estimating the dates of speciation across Apicomplexa is a challenging task, as there are no available fossil documents, whereas the increasing amount of apicomplexan parasite genome data enables estimation of divergence time, which was performed using a Generalized Phylogenetic Coalescent Sampler (G-PhoCS) [22]. *Babesia duncani* and other *Babesia* spp. infective to bovine and ovine species appear to have split around 351.5 million years ago (Mya). Interestingly, one of the *Babesia* species, *B. microti*, derived from a common ancestor with other Piroplasm parasites around 614.2 Mya (Fig. 4). This result is consistent with previous reports that revealed piroplasm parasite speciation events to have been earlier than those of their hosts and vectors [23, 24].

### Babesia duncani species-specific genes

**Fig. 5:**
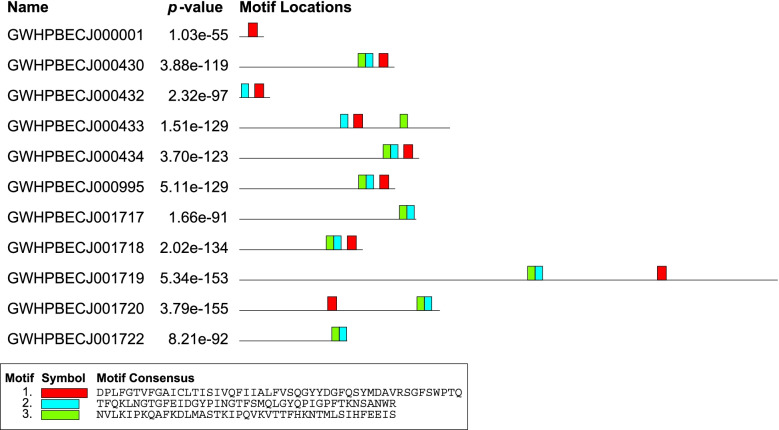
Motif identification of *blf*1. Each colored rectangle represents a different motif. Almost all members in this gene family present at least one of these motifs

**Fig. 6:**
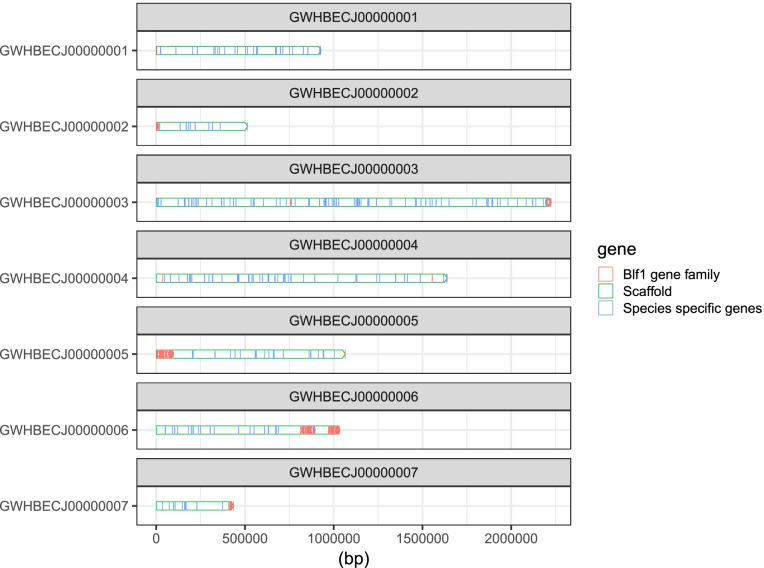
Distributions of 223 *B. duncani*-specific genes and *blf*1 gene family in scaffolds

Multigene families are well known to play critical roles and evolve extremely rapidly during species evolution and adaptation to hosts. Completeness of *B. duncani* genome sequencing facilitates a better understanding of its adaption evolution, vector-pathogen and pathogen-host interactions, and the discovery of virulence factors. For Apicomplexa parasites, most large size of family is species-specific genes, such as *fam* gene families in *Plasmodium gallinaceum* and *Plasmodium relictum*, *var* genes in *P. falciparum*, *pir* genes in *P. vivax*, *and Plasmodium knowlesi*, *msa* in *Babesia* spp. [25–28]. Likewise, we identified the largest gene family in *B. duncani*, containing 89 members that encode a motif conserved across family members, which is a novel family, and is named *B. duncani* largest family 1 (*blf* 1, Additional file 2: Table S2) [29–31]. Additionally, conserved motifs were identified in this gene family by MEME (Fig. 5) [32, 33]. Sixty-five out of 89 genes each encodes a protein with at least one predicted transmembrane helix, and the remainder of these are predicted to be exported. It is impossible to determine significant sequence similarity to other apicomplexan parasite genomes. Almost all of these genes are located in the subtelomeric region, and subtelomeric multigene families in *Plasmodium* spp. have been proved to be important for transporting proteins into/through the host cell (Fig. 6) [34]. RNA-Seq data proved that 53 out of 89 members of *blf* 1 gene family are expressed in blood-stage.

**Fig. 7:**
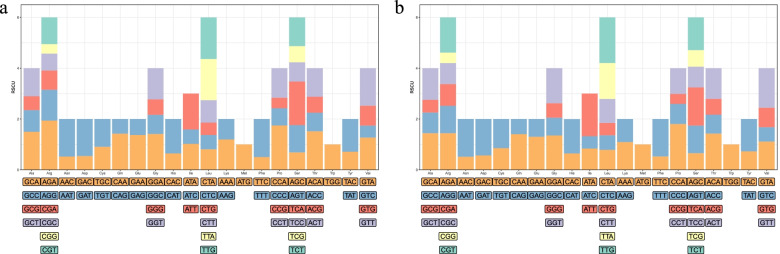
An overall relative synonymous codon usage for 223 species-specific genes and orthologous in *B. duncani*. **a** Relative synonymous codon usage for 223 species-specific genes. **b** Relative synonymous codon usage for orthologous in *B. duncani*

We identify 223 species-specific genes without orthologs in other *Babesia* spp. and *Theileria* spp. included in this study, which were distributed across its whole genome (Fig. 6). Thirty-one of these genes present signal peptide, identified by signalp-5.0b (Additional file 3: Table S3), and almost all proteins encoded by these genes are secreted into the host cell environment using TMHMM, suggesting that these genes might involve in parasite and host/vector receptor interactions [35, 36]. Relative synonymous codon usage was estimated by CodonW v1.4.2 program (https://github.com/smsaladi/codonw-slim). No codon usage bias was observed between 223 species-specific genes and all orthologues in *B. duncani* (Fig. 7). The values of relative synonymous codon usage were tested by paired *t* test, and no significant difference was observed between species-specific genes and all orthologs (*p* = 0.997). Aligning with RNA-Seq data of *B. duncani*, 215 out of 223 *B. duncani* species-specific genes show evidence of expression in the blood-stage, meaning that the remaining eight genes might play a role in other stages of the life cycle. Ninety-eight of these genes are unlikely to ascribe their potential functions by BLASTP and Interproscan (v5.48-83.0), highlighting that limited efforts have been made to explore the content of genome that may play critical roles relating to host specificity and immune evasion [37, 38]. Species-specific genes may be an alternative source of evolutionary innovations and host adaptations, whereas their precise biological functions remain to be investigated.

### Multigene families

**Fig. 8:**
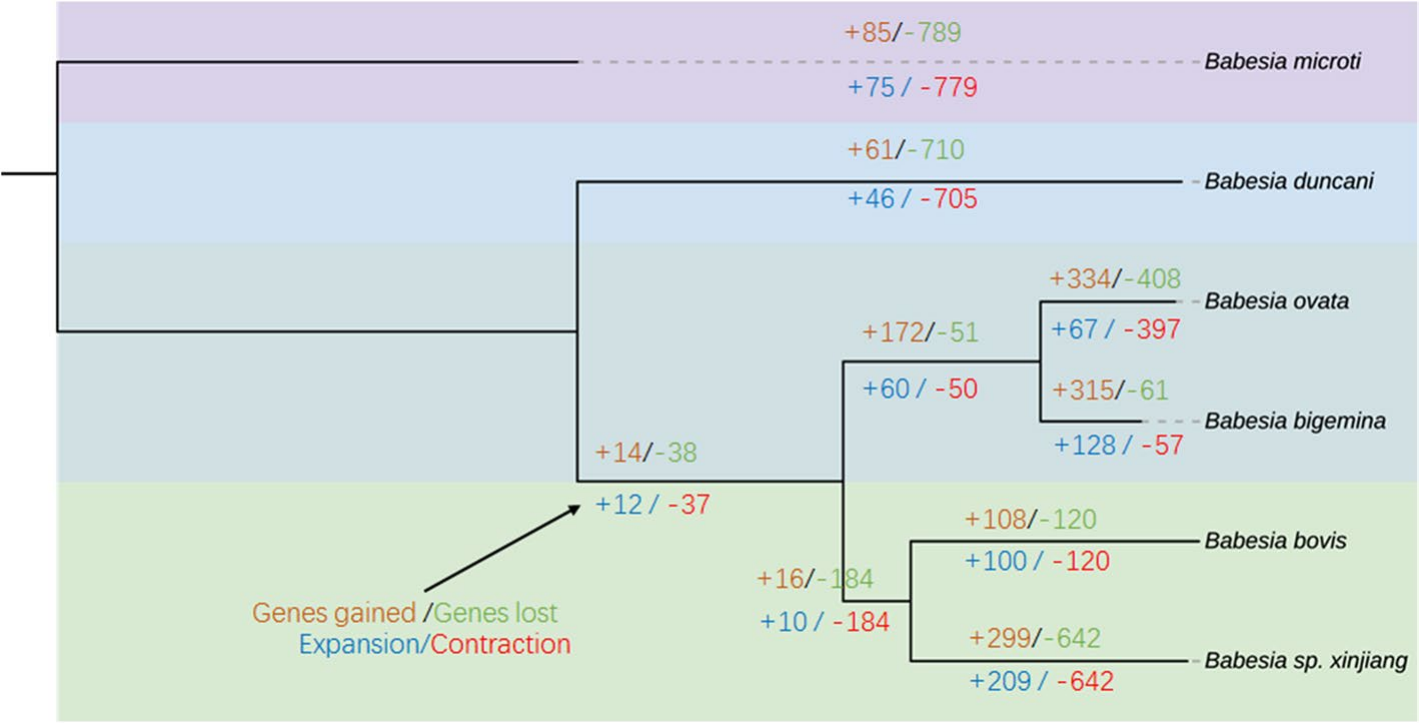
Evolution gene families in *Babesia* spp. The dynamics of gene family sizes in the genomes of *B. microti*, *B. duncani*, *B. ovata*, *B. bigemina*, *B. bovis*, and *Babesia* sp. xinjiang. Numbers below the branches indicate gene family expansions/contractions, and the numbers above the branches show gene gains/losses

Although we observed 46 expanded and 705 contracted gene families involving 61 genes gained and 710 genes lost, except for glycosylphosphatidylinositol-anchored protein family (GPI-AP) and serine esterase (SEA) (Fig. 8; Additional file 5: Table S5), almost all of gene families members slightly increased by 1 or 2 copies and decreased by 1 to 2 copies. Compared with *B. microti*, these expanded and contracted gene families showed no significant difference. These small expansions and contractions may be caused by an artifact of genome sequencing. GPI-APs have been identified in the membranes of apicomplexan, such as *P. falciparum*, *T. gondii*, *B. bovis*, *Trypanosoma brucei*, and *Leishmania donovani* [39–41]. Some of the GPI-APs that bind to receptors of erythrocytes in *B. divergens* and *B. canis* are expressed on the surface of parasite merozoites [42, 43]. *Babesia divergens* antigen Bd37 is a GPI-AP expressed on the surface of merozoites, which has been used to immunize animals against *B. divergens* infection [44]. Subsequently, a homologous GPI-AP of *B. canis* was proved to protect dogs against this parasite infection [45]. These results highlight the feasibility of a more general strategy involving GPI-AP to develop protective vaccines against *Babesia* spp., including *B. duncani* [46]. GPI-APs are also an attractive pool of antigens for vaccine and diagnostic test development. Some of the GPI-APs, including BmGPI12 and BmGPI13 in *B. microti*, and GPI-anchored merozoite surface antigen-1 are highly expressed in red blood cell stages, suggesting their importance for membrane structure or function [47, 48]. BmGPI12 is also a sensitive diagnostic antigen for determining the prevalence of *B. microti* in affected countries [49, 50]. The performance of GPI-APs against *B. duncani* infection and in diagnostic assays needs to be investigated. Concerning the six-cysteine gene family in *P. falciparum*, some members (*Pf48*/*45*, *Pf230*, and *Pf47*) contribute to parasite fertilization, while *PfP52* and *PfP36* perform a vital role in sporozoite invasion of hepatocytes [51–54]. Mosquito stage-specific proteins of the six-cysteine family, such as *P25*, *P28*, *P230*, *P48*/*45*, and *Pfs47*, show significant efficiency in transmission blocking against *Plasmodium*; meanwhile, six-cysteine A and B emphasize candidates from this family blocking *B. bovis* transmission in vector ticks [55–58]. Twenty-three members of this gene family are identified in *B. duncani*. However, whether these members perform similar roles in *B. duncani* development and immune response remains largely unknown.

To complete the life cycle of *Babesia* spp., host cell invasion is initiated with interactions between parasite proteins and host receptors. Thrombospondin-related anonymous proteins (TRAP) contribute to sporozoites of *Plasmodium* infecting salivary glands/live cells and merozoites of *Babesia* infecting red blood cells [59, 60]. In some *Plasmodium* spp., such as *P. berghei* ANKA and *P. vivax* P01, a single copy of TRAP was identified in genomes [60]. However, we identified seven copies in *B. duncani*, revealing that they may contribute to a more sophisticated process in terms of parasite-host interactions. Furthermore, transcriptional evidence of all these genes is found in blood stages, consistent with their role in red blood cell adhesion/invasion.

### *Babesia duncani* adaptive evolution

Using pairwise Ka/Ks comparisons of the *B. duncani* genome with its closest sister species, we are able to discover species-specific adaptation to vectors or hosts. A branch-site model analysis was performed to determine the positive selection across 1437 orthologous genes that occurred in *B. duncani* and other piroplasm parasites. Within the *B. duncani* genome, we identified 38 genes that presented positive selection (Table 2). Furthermore, we determined these positive selection genes to fully examine whether they could perform specific functions in *B. duncani* life cycle. We noticed that some essential genes received selection pressure and took important roles in cellular process, including in transcription (high mobility group protein B1, AP2 domain transcription factor ap2ix-6, helicase), translation (tyrosyl-tRNA synthetase, RNA methyltransferase, ribosomal protein L24, N6-adenine-specific methylase), post-transcription modification (phosphatase methylesterase, GPI ethanolamine phosphate transferase 3), and protein degradation (peptidase, 26S proteasome, ERAD-associated E3 ubiquitin-ligase, ubiquitin carboxyl-terminal hydrolase, ubiquitin carboxyl-terminal hydrolase).

**Table 2:** Positive selection signal present in genes of *B. duncani*

No. of positive selection gene	Protein ID	Protein length (aa)	Predicted protein function
1	GWHPBECJ001716	1815	Predicted protein
2	GWHPBECJ000742	132	Protein DJ-1-like protein B
3	GWHPBECJ001735	105	Uncharacterized protein
4	GWHPBECJ001852	211	Tryptophanyl-tRNA synthetase
5	GWHPBECJ001853	406	Conserved hypothetical protein
6	GWHPBECJ001854	956	DEAD box ATP-dependent RNA helicase family member protein
7	GWHPBECJ001855	386	Coronin
8	GWHPBECJ001856	1032	ATP-dependent helicase rhp16
9	GWHPBECJ001857	108	Ribosomal protein L24 family protein
10	GWHPBECJ001858	87	EF-hand domain-containing protein
11	GWHPBECJ001859	270	Methionine aminopeptidase 1
12	GWHPBECJ001860	284	CD2 antigen cytoplasmic tail-binding 2
13	GWHPBECJ001861	420	Ubiquitin carboxyl-terminal hydrolase
14	GWHPBECJ003724	552	ERAD-associated E3 ubiquitin-ligase
15	GWHPBECJ000199	691	Hypothetical protein, conserved
16	GWHPBECJ003331	969	GPI ethanolamine phosphate transferase 3
17	GWHPBECJ000975	505	Uncharacterized protein
18	GWHPBECJ002933	986	Hypothetical protein
19	GWHPBECJ001102	233	Hypothetical protein
20	GWHPBECJ002272	276	Heme oxygenase (HO)
21	GWHPBECJ002296	250	ABC transporter ATPase
22	GWHPBECJ001213	170	Phosphatase methylesterase
23	GWHPBECJ000505	195	Putative rRNA methyltransferase
24	GWHPBECJ000083	649	Exosome component 10
25	GWHPBECJ001479	361	Hypothetical protein
26	GWHPBECJ000708	460	AP-2 complex subunit alpha-2
27	GWHPBECJ001500	570	Hypothetical protein
28	GWHPBECJ001501	211	High mobility group b1
29	GWHPBECJ001502	1361	Condensin complex subunit 1
30	GWHPBECJ001503	113	Subpellicular microtubule protein 1
31	GWHPBECJ001504	833	5′->3′ exoribonuclease
32	GWHPBECJ001505	318	Hypothetical protein
33	GWHPBECJ001506	376	Tyrosyl-tRNA synthetase
34	GWHPBECJ001507	264	Proteasome subunit alpha
35	GWHPBECJ001508	760	Peptidase, S9A/B/C family, catalytic domain-containing protein
36	GWHPBECJ001509	1084	Helicase SKI2W
37	GWHPBECJ003167	695	Elongation factor G
38	GWHPBECJ001820	76	Secreted ookinete protein

We also observed positive selection events in some genes correlated with the survival environment of *B. duncani*. Selection was detected in a gene involved in taking nutrients from red blood cell plasma (heme oxygenase), implying adaptation to the internal environment of red blood cells. Positive selection was also found in genes contributing to maintaining the morphology of *B. duncani*, including cytoskeleton-associated protein (subpellicular microtubule protein 1) and erythrocyte-binding protein (CD2 antigen cytoplasmic tail-binding 2).

## Conclusions

In conclusion, using ONT sequencing and Illumina sequencing technologies, we assembled and generated the first *B. duncani* reference genome, which is essential to better understand this species’ biological features. We confirmed that *B. duncani* forms a phylogenetically distinct clade from other Piroplasm parasites and estimated the speciation date of *B. duncani* that occurred later than that of *B. microti*, providing new insights into the evolutionary history of *B. duncani*. Two gene families present significant expansion in *B. duncani* and may play important roles in host cell invasion and virulence of *B. duncani*, using comparative genomic analyses. Whether these gene families perform predicted roles needs to be unraveled through genetic manipulation technology and functional studies. Genes identified in *B. duncani* presenting signal of positive selection perform diverse roles in transcription, translation, and post-translated modification processes. Our study provides basic information for further exploring *B. duncani* features, such as host-parasite and tick-parasite interactions.

## Methods

### Sequencing and preparing data

The first case of babesiosis, caused by *B. duncani* WA1, was reported in a 41-year-old man in Washington state. This parasite was obtained from ATCC (PRA-302™) and injected into hamsters. Sub-cloning of this parasite was not performed, as a continuous culture system in vitro has not been developed in our laboratory. When the parasitemia reached 10%, infected red blood cells were collected and merozoites of *B. duncani* were purified as previously reported with minor modification [61]. Briefly, host nucleated blood cells were removed using a syringe filter for white blood cells (PALL, USA). Following this, blood cells were washed three times with cold phosphate-buffered saline (PBS, pH7.4) and lysed by saponin (0.05% in PBS). Merozoites were collected by centrifugation at 10,000*g* for 30 min.

Genomic DNA was extracted using a commercial DNA extractions kit according to the manufacturer’s instructions (QIAamp DNA Blood Mini Kit; Qiagen, Hilden, Germany). The library for PromethION was constructed using a ligation kit (SQK- LSK109, Oxford Nanopore Technology, Oxford, UK) and then analyzed using two FLOMIN106 flow cells (v9.4.1). The raw FAST5 data were base called using Guppy (v3.2.2) [62]. A library of 400-bp paired-end reads of genomic DNA was prepared for genome correction and sequenced using the Illumina sequencing platform. Total RNA was extracted, and library construction was performed according to Illumina TruSeq mRNA library protocol.

### De novo assembly

To remove hamster genomic DNA contamination, the NanoLyse software package was used to compare ONT sequencing data with *Cricetulus griseus* genome (https://www.ncbi.nlm.nih.gov/; accession number GCA_000223135.1) [63]. Eventually, 11,118 reads (account for 5.7% of ONT data) from host genomic DNA were removed. Low-quality reads, contained in ONT sequencing data, were filtered by NanoFilt [63]. Meanwhile, for Illumina sequencing data, low-quality base/reads and adaptor sequences were removed by trim_galore (https://github.com/FelixKrueger/TrimGalore) and contamination of host genome DNA (423,726 paired reads account for 4.49% of Illumina sequencing data) was depleted by aligning Illumina reads with *Cricetulus griseus* genome using bowtie2 [64].

**Fig. 9:**
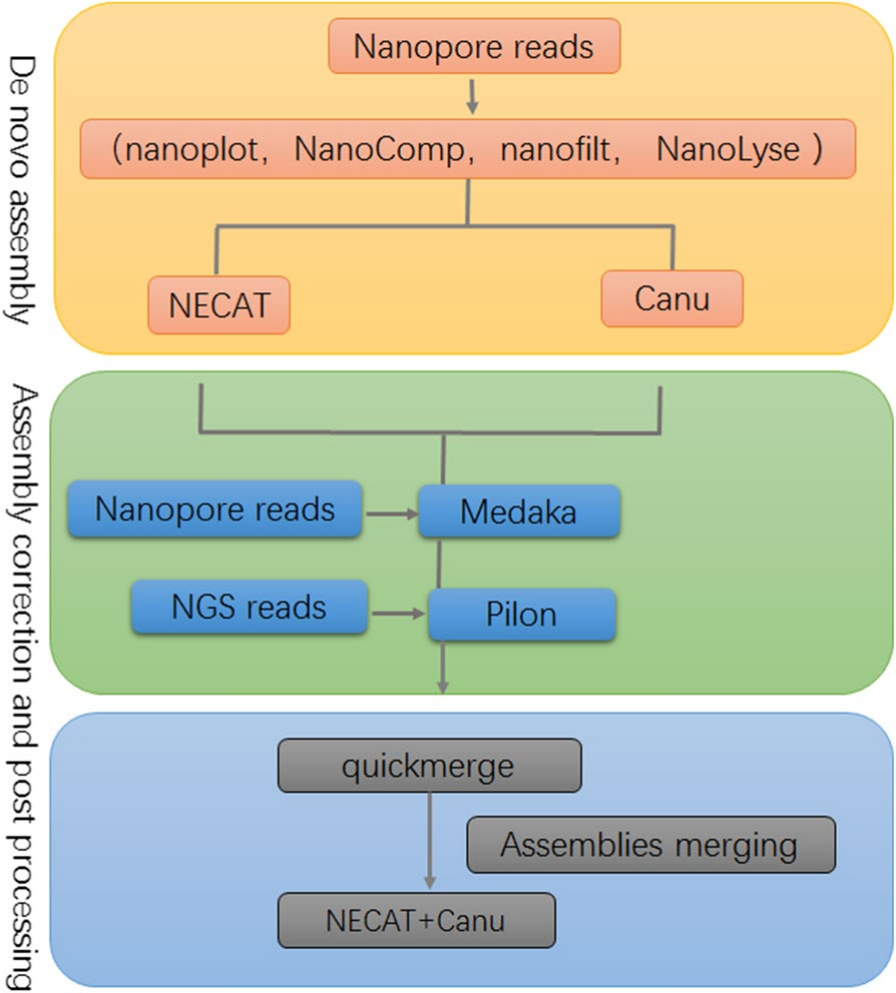
The framework of genome assembly. In the stage of assembly, Nanoplot and NanoComp were applied to statistic reads length and quality distribution. Nanofilt and NanoLyse were employed to remove low-quality reads and remove contaminated DNA from host, respectively. Subsequently, each draft genome was corrected using ONT reads and Illumina reads to improve genome accuracy. Finally, genomes generated from NECAT and Canu were merged with the quickmerge software to produce contiguity assembly

In our previous study, genome assembly pipelines were developed for Piroplasm parasites [65]. Briefly, genome assembly of ONT reads was performed using NECAT (v0.0.1) and Canu (v2.2.2) with default parameters. Correction of raw draft genomes is a critical step in ONT reads assembly (https://ngdc.cncb.ac.cn, CRA004588; https://www.ncbi.nlm.nih.gov/bioproject/PRJNA844476) [66, 67]. Draft genomes were improved by ONT reads self-correction using Medaka (v1.3.4). Further error correction was an essential step using Illumina data, which are available from the National Genomics Data Center (https://ngdc.cncb.ac.cn) under accession number CRA004607 and the National Center for Biotechnology Information (https://www.ncbi.nlm.nih.gov) under accession number PRJNA844476, using Pilon to generate the final assembly output [66–68]. To generate more contiguity assembly, we also merged assembly outputs from assemblies derived from distinct de novo tools (NEACT and Canu) (Fig. 9). Samtools was employed to determine the overall coverage of the genome assembly by mapping Illumina sequencing reads to it. We obtained 98.1% coverage of the assembly. Furthermore, the quality of assembly was evaluated using Benchmarking Universal Single-copy Orthologs (BUSCO v5.1.3) to determine the completeness using the core apicomplexan dataset (apicomplexa_odb10) [20, 69, 70].

### Genome annotation

Before genome annotation, repeat sequences were masked to reduce the requirements of computed resources and to produce reliable annotation outcomes. For this purpose, a standard pipeline was performed including (1) simple tandem repeat sequences predicted using TRF (v4.09) program, (2) ab initio repeat identification using RepeatScout (v1.0.5), and (3) homologous alignment using RepeatMasker program [71–73]. The genome of masked sequences was performed gene structure annotation.

Gene structures were predicted by a combination of ab initio, homology alignment, and transcriptome data. In terms of ab initio, PASA (v2.3.1) was applied to produce candidate gene structures based on the longest open reading frame and a GFF3 file, which could be applied to obtain a set of gene structured for training gene models of Augustus (v3.3.3) and GlimmerHMM (3.0.4) programs [64, 74–77]. Following this, we used Augustus and GlimmerHMM to predict the gene structure based on the trained gene models. Furthermore, ATT, exonerate (2.4.0), and GeneID (v1.4.5) programs were used to align with the UniProt apicomplexan database to identify candidate gene structures [78–80]. Illumina RNA-Seq reads (https://ngdc.cncb.ac.cn, CRA004607) were mapped against the *B. duncani* genome with Tophat2 (v2.1.1) [81]. Mapped reads were processed using Cufflinks (v2.2.1) to generate annotation information as transcriptional prediction data [82–84]. The evidence of gene annotation from ab initio, homologous alignments, and transcriptional data was integrated into a non-redundant gene set by EvidenceModeler (v1.1.1) [85].

### Collinearity analysis

Three species with completed genomes, including *B. duncani* (https://www.cncb.ac.cn/, GWHBECJ00000000), *B. microti* (GCF_000691945.2), and *B. bovis* (AAXT02000000), were selected for collinearity analysis. MCScanX (https://github.com/tanghaibao/jcvi/wiki/MCscan-(Python-version)) was used to identify homologous scaffolds and gene synteny. The pairwise blocks were defined as at least five homologous genes in the 25-gene size window.

### Ortholog group identification and gene family expansion and contraction analysis

Apicomplexan protein sequences were downloaded from NCBI and Plasmo DB databases. The orthologous group across 18 species, including *B. duncani* (https://www.cncb.ac.cn/, GWHBECJ00000000), *B. bigemina* (GCA_000981445.1), *B. bovis* (AAXT02000000), *B. microti* (GCF_000691945.2), *B. ovata* (GCA_002897235.1), *Babesia* sp. Xinjiang (GCA_002095265.1), *T. annulata* (GCA_000003225.1), *T. parva* (GCA_000165365.1), *T. equi* (GCA_000342415.1), *T. orientalis* (GCA_000740895.1), *Toxoplasma gondii* (GCA_000006565.2), *Neospora caninum* (GCA_016097395.1), *Plasmodium falciparum* (GCA_000002765.3), *P. vivax* (GCA_000002415.2), *P. yoelii* (GCA_900002385.2), *Cryptosporidium parvum* (GCA_000165345.1), *Eimeria tenella* (GCA_000499545.1), and *Tetrahymena thermophila* (GCA_000189635.1), were identified using OrthoFinder (v2.5.4), which is a practical, fast, accurate, and comprehensive tool for comparative genomes [86, 87]. The program, based on amino acid sequence alignment, uses diamond, and the important parameter inflation index was set at 1.5 to balance sensitivity and selectivity. Identified ortholog groups were used for further analysis of gene family expansion and contraction with café (v2.0) [88, 89].

### Phylogenetic analysis and divergence time estimation

Three hundred ten single-copy orthologous that were present in 18 species of Apicomplexa parasites were aligned with MUSCLE, and ambiguous alignments were processed using Gblocks with default parameters (parameters: -t = p –b = h –p =n –b4 = 2) [90]. Then aligned sequences were concatenated by custom scripts to generate FASTA files for further phylogenetic analysis. The maximum likelihood phylogenetic trees were generated by RAxML with the best fit model LG+F+R5 [91]. ITOL was used to visualize and edit the labels of phylogenetic trees (http://itol2.embl.de/upload.cgi) [92].

The divergence time for Apicomplexa parasites was estimated using the mcmctree program with three correlated time points, 817 million years ago (Mya, divergence time between *T. gondii* and *P. falciparum*, ranging from 580 to 817 Mya), 470 Mya (divergence time between *E. tenella* and *N. caninum*), and 1290 Mya (divergence time between *C. parvum* and *T. thermophila*, ranging from 767 to 1344 Mya) [93–97].

### Ka/Ks analysis

The nonsynonymous (Ka) and synonymous (Ks) substitution rates and positive selection strength (Ka/Ks) were calculated by KaKs_Calculator (v2.0) [98]. First, reciprocal BLAST was used to run the pairwise alignments between *Babesia* spp., the *e*-value was set to 10^−5^, and the number of hits for each pair of species was set to 5. Second, each pairwise protein sequence was aligned by MUSCLE, and pairwise nucleotide sequence alignments were generated by transforming protein alignments into codon alignments with ParaAT [99]. Third, Ka/Ks ratios were calculated based on pairwise codon alignments using KaKs_Calculator, and the models of KaKs_Calculator were invoked from PAML. M0 model (Branch site model) was used in this study [100].

## Supplementary Information

**Additional file 1: Table S1**. Protein annotations of *B. duncani* using BLASTP and Interproscan programs.

**Additional file 2: Table S2**. *Babesia duncani* largest gene family 1.

**Additional file 3: Table S3**. 223 species specific genes in *B. duncani*.

**Additional file 4: Table S4**. Multi-gene families in *B. duncani.*

**Additional file 5: Table S5**. Significantly expanded gene families in *B. duncani*.

## Data Availability

All data generated or analyzed during this study are included in the article, its supplementary information files are publicly available in repositories. The genomic sequencing data of ONT reads and Illumina reads presented in this study have been deposited in the China National Center for Bioinformation (https://www.cncb.ac.cn/; CRA004588 and CRA004607) and the NCBI Sequence Read Archive (SRA) database under project accession number PRJNA844476 [66, 67]. The RNA-seq data have also been deposited in the China National Center for Bioinformation (CRA004607) and the NCBI Sequence Read Archive (SRA) database under project accession number PRJNA84447 [66, 67].

## Abbreviations

ONT: Oxford Nanopore Technology
ITS: Internal transcribed spacer
GO: Gene Ontology
*CoxI*: Coxidase subunit I
*Cob*: Cytochrome b
*blf* 1: *B. duncani* largest family 1
TRAP: Thrombospondin-related anonymous protein
GPI-AP: Glycosylphosphatidylinositol-anchored protein family
SEA: Serine esterase
PBS: Phosphate-buffered saline
Ka: Nonsynonymous
Ks: Synonymous
